# Impact of Tumor Regression Grade as a Major Prognostic Factor in Locally Advanced Rectal Cancer after Neoadjuvant Chemoradiotherapy: A Proposal for a Modified Staging System

**DOI:** 10.3390/cancers10090319

**Published:** 2018-09-07

**Authors:** Changhoon Song, Joo-Hyun Chung, Sung-Bum Kang, Duck-Woo Kim, Heung-Kwon Oh, Hye Seung Lee, Jin Won Kim, Keun-Wook Lee, Jee Hyun Kim, Jae-Sung Kim

**Affiliations:** 1 Department of Radiation Oncology, Seoul National University College of Medicine, Seoul National University Bundang Hospital, 82 Gumi-ro 173beon-gil, Bundang-gu, Seongnam 13620, Korea; songc@snubh.org (C.S.); gag920@naver.com (J.-H.C.); 2 Department of Surgery, Seoul National University College of Medicine, Seoul National University Bundang Hospital, 82 Gumi-ro 173beon-gil, Bundang-gu, Seongnam 13620, Korea; kangsb@snubh.org (S.-B.K.); kdw@snubh.org (D.-W.K.); crsohk@snubh.org (H.-K.O.); 3 Department of Pathology, Seoul National University College of Medicine, Seoul National University Bundang Hospital, 82 Gumi-ro 173beon-gil, Bundang-gu, Seongnam 13620, Korea; mw9195@snubh.org; 4 Department of Internal Medicine, Seoul National University College of Medicine, Seoul National University Bundang Hospital, 82 Gumi-ro 173beon-gil, Bundang-gu, Seongnam 13620, Korea; jwkim@snubh.org (J.W.K.); hmdoctor@snubh.org (K.-W.L.); jhkimmd@snubh.org (J.H.K.)

**Keywords:** tumor regression grade, neoadjuvant chemoradiotherapy, rectal cancer, biomarkers

## Abstract

There is ongoing debate regarding the significance of complete or near-complete response after neoadjuvant chemoradiotherapy (CRT) for rectal cancer. This study assessed the prognostic value of the Dworak tumor regression grade (TRG) following neoadjuvant CRT and surgery primarily in patients with pathological stage (ypStage) II and III rectal cancer. The records of 331 patients who underwent neoadjuvant CRT followed by total mesorectal excision between 2004 and 2015 were retrospectively reviewed. Patients were categorized as having a good response (GR, TRG 3/4, *n* = 122) or a poor response (PR, TRG 1/2, *n* = 209). At a median follow-up of 65 months, five-year disease-free survival (DFS) was higher in the GR group than in the PR group (91.3% vs. 66.6%, *p* < 0.001). Patients with a GR and ypStage II disease had a five-year DFS that was indistinguishable from that of patients with ypStage 0–I disease (92.3% vs. 90.7%, *p* = 0.885). Likewise, patients with a GR and ypStage III disease had a five-year DFS similar to those with ypStage II disease (76.0% vs. 75.9%, *p* = 0.789). A new modified staging system that incorporates grouped TRG (GR vs. PR) was developed. The prognostic performance of this modified stage and the ypStage was compared with the Harrell C statistic. C statistic of the modified stage was higher than that of the ypStage (0.784 vs. 0.757, *p* = 0.012). The results remained robust after multivariate Cox regression analyses. In conclusion, a GR to neoadjuvant CRT is an independent predictor of good DFS and overall survival and further stratifies patients so as to estimate the risk of recurrence and survival among patients with ypStage II and III rectal cancer.

## 1. Introduction

Neoadjuvant chemoradiotherapy (CRT) followed by total mesorectal excision (TME) is currently the standard management for clinically staged, locally advanced rectal cancer. The German Rectal Cancer Study Group trial demonstrated that this approach improved local control and sphincter preservation and reduced toxicity [1]. 

The tumor response to neoadjuvant CRT reflects the underlying tumor biology and might be used as a surrogate for treatment outcome [2,3,4,5]. In the resected specimen, histological changes caused by CRT in the primary tumor are assessed with the tumor regression grade (TRG), a five-tier grading system initially described by Dworak et al. [6]. The grading of Dworak TRG system is defined as follows: 0, no regression; 1, dominant tumor mass with obvious fibrosis and/or vasculopathy; 2, dominant fibrotic changes with few tumor cells or groups (easy to find); 3, very few (difficult to find microscopically) tumor cells in fibrotic tissue with or without mucous substance; and 4, no tumor cells and only a fibrotic mass (total regression or response). The TRG ranges from a pathological complete response (pCR) to no tumor regression at all [7]. Several studies have shown that patients with pCR have more favorable outcomes than those of patients without pCR in terms of local control, distant metastases, disease-free survival (DFS), and overall survival (OS) [8,9,10]. However, pCR is present in only 8–20% of patients [1,11], raising the question as to whether a more precise stratification with TRGs could serve as a prognostic factor for a wider range of patients. Including patients with a partial or near-complete response along with those with pCR to define good responders may help identify additional patients with better outcomes and differentiate them from those with an increased risk of disease recurrence. Although several studies have evaluated the prognostic significance of partial or near-complete response, the results are conflicting [8,12,13,14,15,16,17,18,19,20]. So far, only a few studies have examined the potential complementary value of TRG with the American Joint Committee on Cancer (AJCC) TNM pathologic staging system in predicting recurrence or survival [7,21,22,23]. 

We hypothesized that complete or near-complete response to neoadjuvant CRT is associated with a better clinical outcome among patients within specific pathological stages. In the present study of patients with rectal cancer who underwent neoadjuvant CRT, we aimed to evaluate the prognostic value of the Dworak TRG (grouped 3/4 vs. 1/2) primarily for patients with pathological stage II and III rectal cancer and to compare the prognostic performance of the 7th edition of the AJCC TNM pathologic staging with a new modified staging system that incorporates the Dworak TRG grouping.

## 2. Results

### 2.1. Patient Population 

The median patient age at the time of neoadjuvant CRT was 61.2 years (range 28.2–82.7 years); 69.2% were men. The median tumor distance from the anal verge was 5 cm (interquartile range (IQR), 4–6 cm). A majority of tumors were cT3 on preoperative evaluation (*n* = 278; 84.0%), and 281 patients (84.9%) had clinical lymph node involvement. After proctectomy, the pathological stage following neoadjuvant CRT (ypStage) was 0 in 45 patients (13.6%), I in 94 (28.4%), II in 80 (24.2%), and III in 112 (33.8%). Sphincter-preserving surgery was performed for 293 patients (88.5%). A total of 23 (6.9%) had pathological circumferential resection margin (CRM) involvement. Following radical proctectomy, 289 (87.3%) patients received 5-FU–based adjuvant chemotherapy. 

The median follow-up was 65.0 months (range, 8.4–159.3 months). Locoregional recurrence developed in 25 patients (7.5%) and distant metastases in 68 (20.5%), of whom 24 had both locoregional recurrence and distant metastases. The five-year local control and five-year distant metastasis-free survival rates were 91.7% and 79.2%, respectively. The five-year OS and five-year DFS rates were 86.9% and 75.7%, respectively.

### 2.2. Patient and Tumor Characteristics by TRG Grouping 

Among the 331 patients, 50 (15.1%) had TRG 4, 72 (21.8%) TRG 3, 154 (46.5%) TRG 2, and 55 (16.6%) TRG 1. None had TRG 0. TRG 4 was observed in 30% of those with cT2, 14.7% with cT3, and 9.1% with cT4 tumors.

There were 122 (36.9%) patients with a good response (GR, TRG 3/4) and 209 (63.1%) with a PR (PR, TRG 1/2; Table 1). In patients with a PR, lymphatic, venous, and perineural invasion as well as CRM involvement were more common, and the cT, ypT, and ypN designations were higher. Abdominoperineal resection was more frequently performed for patients who had a PR. A higher proportion of patients with a PR had an interval shorter than 42 days between completion of CRT and surgery.

### 2.3. TRG as a Prognostic Factor for DFS

**Figure 1:**
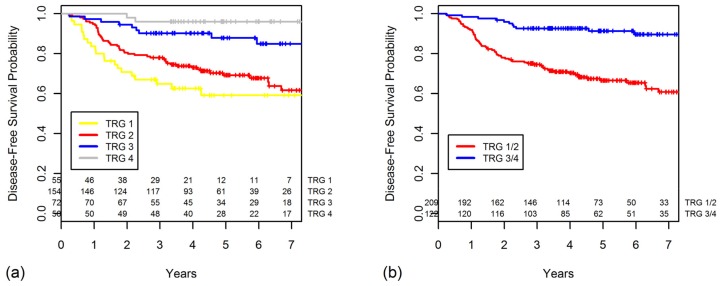
Disease-free survival (DFS) curves are shown for categories of (**a**) tumor gregression grade (TRG) and (**b**) grouped TRG.

The five-year DFS progressively worsened with lower TRGs (TRG 4, 96.0%; 3, 87.8%; 2, 69.2%; and 1, 59.1%) (Figure 1a). There were no statistically significant differences in DFS between the TRG 3 and TRG 4 groups (*p* = 0.068), but the TRG 3 group had a significantly better DFS than that of the TRG 2 group (*p* = 0.002). The five-year DFS was significantly higher in the GR group than in the PR group (*p* < 0.001, Figure 1b).

**Figure 2:**
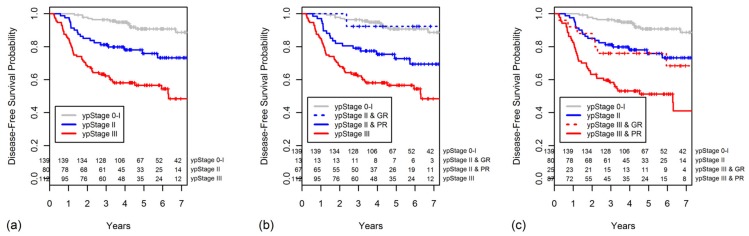
Disease-free survival curves are shown for categories of (**a**) ypStage, (**b**) ypStage II group was dichotomized as good response (GR) or poor response (PR), and (**c**) ypStage III group was dichotomized as GR or PR.

The five-year DFS rates according to the ypStage also worsened as the stage increased (ypStage 0, 97.8%; I, 87.0%; II, 75.9%; III, 56.6%; *p* < 0.001). The five-year DFS rate for combined ypStages 0–I was 90.7% (Figure 2a). When the ypStage II group was dichotomized by response based on the TRG (Figure 2b), the GR ypStage II group had somewhat, but not significantly, better five-year DFS in comparison to the PR ypStage II group (92.3% vs. 72.8%, *p* = 0.110). The DFS of patients with GR ypStage II was indistinguishable from that of patients with ypStage 0–I disease (*p* = 0.885).

Likewise, when the ypStage III group was dichotomized by response according to the TRG (Figure 2c), the GR ypStage III group had a significantly better five-year DFS in comparison to the PR ypStage III group (76.0% vs. 51.3%, *p* = 0.040). The GR ypStage III group had a DFS similar to that of the ypStage II group (*p* = 0.789).

Other factors that significantly correlated with DFS by univariate analysis included the cT and cN classification; lymphatic, venous, and perineural invasion; ypT and ypN classification; ypStage; and CRM involvement (Table 2). On multivariate analysis, ypN classification, perineural invasion, and TRG remained significant predictors of DFS (Table 3).

### 2.4. TRG as a Prognostic Factor for OS

**Figure 3:**
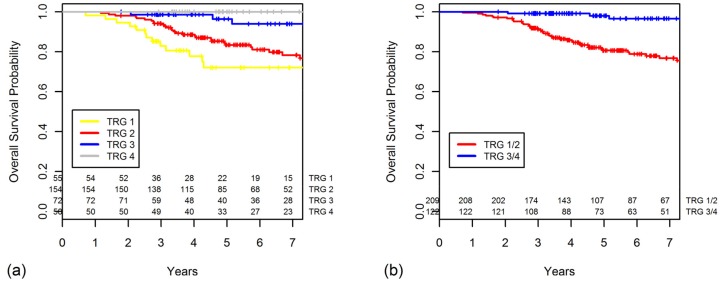
Overall survival (OS) curves are shown for categories of (**a**) TRG and (**b**) grouped TRG.

The five-year OS rate worsened as the TRG decreased (TRG 4, 100%; 3, 96.4%; 2, 83.4%; 1, 72.0%; Figure 3a). The OS was significantly higher for patients with a GR than for those with a PR (*p* < 0.001, Figure 3b).

**Figure 4:**
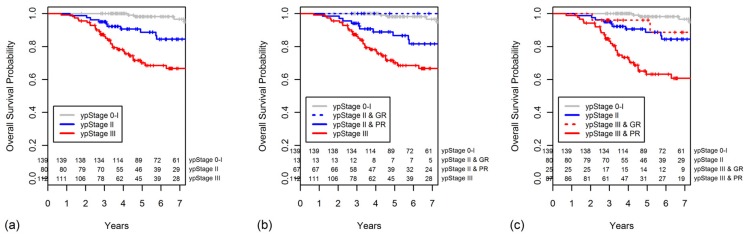
Overall survival curves are shown for categories of (**a**) ypStage, (**b**) ypStage II group was dichotomized as GR or PR, and (**c**) ypStage III group was dichotomized as GR or PR.

The five-year OS also decreased with increasing ypStage (0–I, 98.1%; II, 88.7%; III, 70.1%; *p* < 0.001, Figure 4a). When the ypStage II group was dichotomized by response according to the TRG, the GR ypStage II group had a better, although not significantly, five-year OS in comparison to the PR ypStage II group (100% vs. 86.6%, *p* = 0.120). The OS of patients with GR ypStage II was indistinguishable from that of patients with ypStage 0–I disease (*p* = 0.513). However, the OS of patients with PR ypStage II was similar to that of patients with ypStage III disease (*p* = 0.072). The survival curves for this classification are shown in Figure 4b. 

When the ypStage III group was dichotomized as GR or PR, the GR ypStage III group had a significantly better OS compared with that of the PR ypStage III group (96.0% vs. 63.2%, *p* = 0.019, Figure 4c). The GR ypStage III had an OS similar to that of the ypStage II group (*p* = 0.444, Figure 4c). 

Other factors that significantly correlated with OS by univariate analysis included age; lymphatic, venous, and perineural invasion; ypT and ypN classification; ypStage; and CRM involvement (Table 2). On multivariate analysis, age, ypN classification, perineural invasion, and TRG remained significant predictors of OS (Table 3).

### 2.5. Constructing a New Modified Staging System That Combines ypStage and Grouped TRG 

To further confirm the prognostic value of TRG, we developed a new modified staging system that combines the 7th edition of the AJCC ypStage and grouped TRG (TRG 1/2 vs. 3/4). The new modified staging system is as follows: modified Stage 0, ypStage 0-I and TRG 3/4 or ypStage II and TRG3/4; modified Stage I, ypStage 0-I and TRG 1/2; modified Stage II, ypStage II and TRG 1/2 or ypStage III and TRG 3/4; and modified Stage III, ypStage III and TRG1/2 (Table 4).

**Figure 5:**
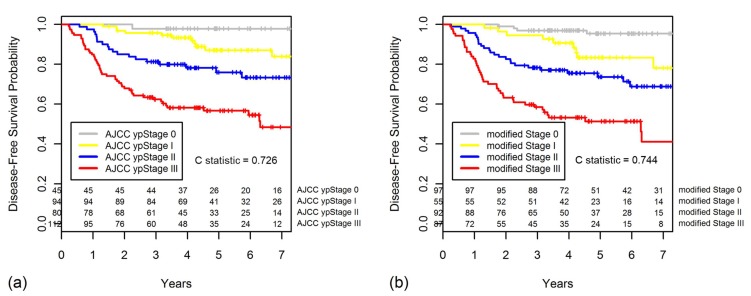
Kaplan-Meier curves for DFS comparing (**a**) the American Joint Committee on Cancer (AJCC) ypStaging and (**b**) the new modified staging system.

The Kaplan-Meier curves for DFS regarding ypStaging and the new modified staging system are shown in the Figure 5. For the new modified staging system, 97 patients were in the modified Stage 0 with 95.3% five-year DFS, 55 in Stage I (83.3%), 92 in stage II (73.6%), and 87 in Stage III (51.3%).

Compared with the AJCC ypStaging system, the new modified staging had better discriminatory ability for DFS, with greater Harrell C statistic (0.744 vs. 0.726), although the difference was not statistically significant (*p* = 0.108). This finding implies that the new staging system that incorporated the Dworak TRG (grouped 1/2 vs. 3/4) provided a better classification system for locally advanced rectal cancer than the 7th edition of the AJCC TNM staging system.

**Figure 6:**
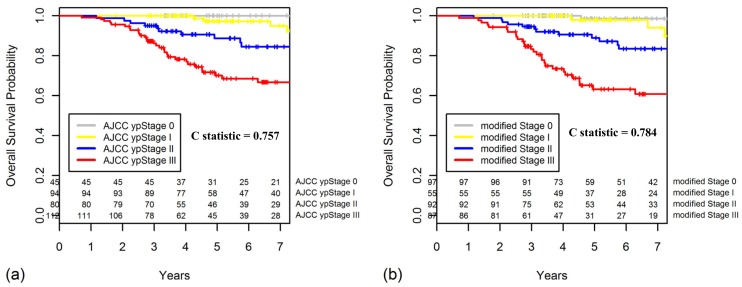
Kaplan-Meier curves for OS comparing (**a**) the AJCC ypStaging and (**b**) the new modified staging system.

The five-year OS also decreased with increasing modified Stage (0, 98.6%; I, 97.9%, II, 88.9%; III, 63.2%; *p* < 0.001). The Kaplan-Meier curves for OS regarding the AJCC ypStaging and the new modified staging system are shown in Figure 6. Compared with the AJCC ypStaging system, the new modified staging had better discriminatory ability for OS, with greater Harrell C statistic (0.784 vs. 0.757, *p* = 0.012).

## 3. Discussion

This study demonstrated that the Dworak TRG system is an independent prognostic factor for recurrence and survival in patients with locally advanced rectal cancer treated with neoadjuvant CRT followed by TME. Even after adjusting for other well-established prognostic factors, such as ypN classification by multivariate analysis, the prognostic value of TRG remained significant. Furthermore, we observed that both complete response (TRG 4) and near-complete response (TRG 3) were associated with five-year recurrence and survival rates significantly different than TRG 1 or 2. Furthermore, among patients with ypStage II or III disease, a GR (TRG 3/4) indicated a better prognosis than a PR (TRG 1/2).

In contrast to pCR, which has a single definition with no visible microscopic disease in both the primary tumor and lymph nodes, various grading systems are used for TRG: Mandard (five, three-tier), Dworak (five-tier), Dworak/Rödel (five, three-tier), Memorial Sloan Kettering Cancer Center (MSKCC) (three-tier), and AJCC/College of Pathology (CAP) system (four-tier) [24]. Although several studies have evaluated the prognostic significance of partial or near-complete tumor regression after neoadjuvant CRT, the results were conflicting [8,12,13,14,15,19,20]. The use of different grading systems, different endpoints for partial or near-complete response, heterogeneous treatment strategies, various intervals between the end of CRT and surgery, and the lack of pathology quality control have made the results challenging to interpret [25]. 

In the most conclusive data from German prospective trials, they have shown the prognostic value of the three-tier Dworak/Rödel TRG system for DFS, OS, and local control [8,17,26]. However, TRG 3 and TRG 2 were grouped together and compared with TRG 4, obscuring the prognostic significance of TRG 3. The definition of Dworak/Rödel TRG 3 in German studies was “regression of >50% of the tumor mass” and is different from that of the original Dworak TRG 3 in the current study, which is “very few (difficult to find microscopically) tumor cells in fibrotic tissue with or without mucous substance.”

One of the most important issues with the Dworak TRG system was interpretation of “difficult to find microscopically” and “easy to find,” which are used to distinguish TRG 3 from TRG 2. Although it is assumed that “difficult to find” means tumor cells found only after assiduous high-power search, significant inter- and intraobserver variability exists [25]. All the other TRG systems including Mandard, Dworak/Rödel, MSKCC, and AJCC/CAP also showed a low concordance rate, indicating poor reproducibility of these systems [7,24,27]. Thus, imaging modalities like magnetic resonance imaging or molecular biomarkers have been studied for assessment of tumor response to neoadjuvant CRT [28,29]. Integrating diverse types of biomarkers such as TRG and other clinicopathological and imaging features could improve the predictive accuracy in patients with locally advanced rectal cancer.

Meanwhile, the role of adjuvant chemotherapy in patients with rectal cancer who have received neoadjuvant therapy remains controversial. Four randomized clinical trials have been published to date comparing adjuvant chemotherapy with no adjuvant treatment after initial neoadjuvant radiotherapy or CRT followed by TME for cT3 to 4 or cN+ rectal cancer [30,31,32,33]. Although all these trials were underpowered to detect small survival benefits, they all concluded that adjuvant chemotherapy yielded no survival benefit in this setting. However, many clinicians still expect that a subset of patients will benefit from adjuvant chemotherapy. Indeed, initial subgroup analysis from the European Organization for Research and Treatment of Cancer 22921 trial suggested that patients benefitted from adjuvant fluorouracil and leucovorin only if they had ypT0–2 after neoadjuvant CRT [34], although DFS and OS were reportedly not improved on long-term analysis [33]. In the ADORE trial, where patients with ypStage II and III disease were randomized to four cycles of fluorouracil and leucovorin versus eight cycles of fluorouracil, leucovorin, and oxaliplatin (FOLFOX) after neoadjuvant CRT and TME, there was significant improvement in the three-year DFS in favor of FOLFOX only for patients with ypStage III disease, suggesting that patients with disease that is less responsive to fluorouracil-based neoadjuvant CRT might benefit from the addition of oxaliplatin to the adjuvant chemotherapy regimen [35]. However, for patients with ypStage II disease, there was no significant difference in the three-year DFS between the two regimens. This might be explained by the heterogeneous characteristics of patients with ypStage II disease, comprising both patients with initial cT3–4N0 disease unresponsive to fluorouracil-based neoadjuvant CRT and those with initial cN+ disease that had responded to treatment. 

In the current study we have showed that patients with a GR and ypStage II disease had a five-year DFS that was indistinguishable from that of patients with ypStage 0–I disease. Likewise, patients with a GR and ypStage III disease had a five-year DFS similar to those with ypStage II disease. These observations also represent a distinct population within the same ypStage and suggest that the identification of tumor heterogeneity may be promoted by neoadjuvant CRT. The incorporation of grouped TRG into the ypStaging system may improve the identification of patients who would benefit the most from adjuvant chemotherapy, and it can be used for treatment stratification for future trials. For example, among patients with ypStage III who had a TRG-defined PR, intensive FOLFOX chemotherapy might be considered in the adjuvant setting. In contrast, patients with a TRG-defined GR and ypStage III might not need such an intensive regimen as FOLFOX. Likewise, patients with ypStage II disease who had had a TRG-defined GR might not need further adjuvant chemotherapy, thus avoiding unnecessary adverse drug effects.

Owing to the retrospective nature of this study, there were certain inherent limitations. First, there was potential for bias due to loss to follow-up. Second, although our TRG classification was based on the Dworak system, there is no universal consensus on the classification of tumor regression after neoadjuvant therapy. Third, the number of patients who had ypStage II disease and a GR was quite small (*n* = 13), thus limiting the conclusions that can be drawn from our results in such patients.

## 4. Materials and Methods 

This study retrospectively analyzed records from a database of patients who underwent rectal cancer surgery after neoadjuvant CRT for locally advanced rectal cancer at our institution between February 2004 and August 2015. The study inclusion criteria were (1) histologically confirmed primary middle or low rectal cancer (within 10 cm above the anal verge); (2) locally advanced resectable disease defined as cT3/T4 or lymph node involvement based on magnetic resonance imaging, with or without transrectal ultrasonography; (3) low-lying early stage disease requiring abdominoperineal resection; (4) no evidence of distant metastasis or concurrent malignancy on pretreatment workup; (5) completion of neoadjuvant of CRT; and (6) TME. A total of 331 patients met these criteria, and their outcomes were analyzed. The study protocol was approved by the institutional review board of Seoul National University Bundang Hospital (B-1705/396-105), and the requirement for informed consent was waived due to the retrospective study design.

All patients underwent neoadjuvant CRT as previously described [36,37]. In brief, long-course radiotherapy was administered over 5.5 weeks at a dose of 50.4 Gy, of which 45 Gy was applied in 25 fractions to the pelvis and a 5.4 Gy boost in 3 fractions to the primary tumor. One of two concurrent chemotherapeutic regimens during radiotherapy was used: continuous oral administration of capecitabine (825 mg/m^2^ twice daily) in 210 patients and florouracil and leucovorin (2 cycles of monthly intravenous bolus of fluorouracil (400 mg/m^2^/day, days 1–3) and leucovorin (20 mg/m^2^/day, days 1–3)) in 115 patients. The remaining 6 patients received a combination regimen, including cetuximab, irinotecan, and capecitabine, as part of a clinical trial. After completion of neoadjuvant CRT, all patients underwent TME. The median interval from the completion of CRT to TME was 47 days (IQR, 43–51 days). Adjuvant chemotherapy was recommended after resection for all patients who were medically fit.

After resection, the specimen was meticulously examined, and the entire tumor bed was embedded for hematoxylin-and-eosin staining and microscopic evaluation. Pathological tumor response to neoadjuvant CRT was scored by using the criteria developed by Dworak et al. [6]. The Dworak regression grading was adopted as the standard pathology reporting protocol by our institution in April 2004, however, both Dworak and AJCC/CAP regression grading have been used as the standard pathology reporting protocol starting from February 2010. For 94% of patients in the current study, the tumor regression grading was determined by one of three dedicated GI pathologists (61% by H.S.L, 25% by E.S, and 8% by H.E.L). The Dworak TRG used in the current study was determined by chart review from our electronic medical record [38]. We did not re-evaluate the Dworak TRG for the current study because the reports were verified by dedicated GI pathologists according to the standard reporting protocol at the time when the reports were produced. 

Based on the findings from previous studies [12,13,15,39,40], patients were categorized as having a GR (TRG 3 or 4) or a PR (TRG 1 or 2) to neoadjuvant CRT. CRM involvement was considered positive if a microscopic tumor was found ≤1 mm from the resection margin [41,42]. Clinical and pathological staging were determined according to the AJCC TNM staging system, 7th edition [43]. For further analysis, we combined ypStages 0 and I, as was done in previous studies [44,45,46].

Continuous variables were evaluated by the Student’s t-test, while categorical variables were compared by the chi-square test. DFS and OS curves were calculated using the Kaplan–Meier method, and differences between curves were assessed with the log-rank test. Factors initially significant on univariate analysis (*p* < 0.10) were entered into a multivariate Cox proportional hazards model and backward stepwise elimination with a threshold of *p* = 0.10 was used to select factors in the final model. The prognostic strength of the AJCC TNM ypStaging and new modified staging system were assessed with the Harrell C statistic. The Harrell C statistic indicated model prediction as follows: 0.5, equal chance; 0.7 to 0.8, acceptable; 0.8 to 0.9, excellent; and 0.9 to 1, outstanding. Statistical analysis was performed by using R software (version 3.3.3; R Foundation for Statistical Computing, Vienna, Austria). All *p* values reported are two-sided, with *p* < 0.05 used to denote statistical significance.

## 5. Conclusions

In summary, our study demonstrates that a TRG-defined GR to neoadjuvant CRT is an independent predictor of good DFS and OS in patients with rectal cancer. When assessing prognosis after neoadjuvant treatment and TME, adding the Dworak TRG system further stratifies patients with ypStage II and III disease in terms of risk of recurrence and survival. Furthermore, the modified staging system that developed in the current study had better OS and DFS discriminatory ability than the 7th edition of AJCC TNM pathologic staging system and may improve the identification of patients who would benefit the most from adjuvant chemotherapy.

## Figures and Tables

**Table 1:** Clinicopathologic characteristics of patients with a good response (GR, TRG 3/4) and poor response (PR, TRG 1/2).

Variable	GR (*n* = 122)	PR (*n* = 209)	*p* Value
Age at diagnosis			0.404
≤61 years	63 (51.6%)	98 (46.9%)	
>61 years	59 (48.4%)	111 (53.1%)	
Sex			0.553
Male	82 (67.2%)	147 (70.3%)	
Female	40 (32.8%)	62 (29.7%)	
Clinical T classification			**0.018**
cT2	12 (9.8%)	8 (3.8%)	
cT3	103 (84.4%)	175 (83.7%)	
cT4	7 (5.7%)	26 (12.4%)	
Clinical N classification			0.256
cN0	22 (18.0%)	28 (13.4%)	
cN+	100 (82.0%)	181 (86.6%)	
Distance from anal verge			0.747
≤5 cm	74 (60.7%)	123 (58.9%)	
>5 cm	48 (39.3%)	86 (41.1%)	
Type of surgery			**0.012**
Sphincter-preserving surgery	115 (94.3%)	178 (85.2%)	
Abdominoperineal resection	7 (5.7%)	31 (14.8%)	
Interval completion of CRT to surgery			**0.029**
<42 days	7 (5.7%)	28 (13.4%)	
≥42 days	115 (94.3%)	181 (86.6%)	
Adjuvant chemotherapy			0.396
No	13 (10.7%)	29 (13.9%)	
Yes	109 (89.3%)	180 (86.1%)	
Regimen of adjuvant chemotherapy			**0.027**
Single agent	88 (80.7%)	124 (68.9%)	
Combination	21 (19.3%)	56 (31.1%)	
Lymphatic invasion			**0.007**
No	115 (95.8%)	181 (86.6%)	
Yes	5 (4.2%)	28 (13.4%)	
Venous invasion			**0.001**
No	118 (98.3%)	184 (88.0%)	
Yes	2 (1.7%)	25 (12.0%)	
Perineural invasion			**<0.001**
No	114 (95.0%)	148 (70.8%)	
Yes	6 (5.0%)	61 (29.2%)	
ypT classification			**<0.001**
ypT0	50 (41.0%)	0 (0%)	
ypT1	14 (11.5%)	8 (3.8%)	
ypT2	31 (25.4%)	60 (28.7%)	
ypT3	26 (21.3%)	138 (66.0%)	
ypT4	1 (0.8%)	3 (1.4%)	
ypN classification			**<0.001**
ypN0	97 (79.5%)	122 (58.4%)	
ypN1	18 (14.8%)	69 (33.0%)	
ypN2	7 (5.7%)	18 (8.6%)	
ypTN classification			**<0.001**
ypT0N0	45 (36.9%)	0 (0%)	
ypT1–2N0	39 (32.0%)	55 (26.3%)	
ypT3–4N0	13 (10.7%)	67 (32.1%)	
ypT0N+	5 (4.1%)	0 (0%)	
ypT1–2N1	6 (4.9%)	13 (6.2%)	
ypT3–4N1	10 (8.2%)	56 (26.8%)	
ypT1–4N2	4 (3.3%)	18 (8.6%)	
Number of harvested lymph nodes ^a^			
All patients	25 (20–31)	24 (18–34)	0.813
ypStage 0	25 (20–30)	–	–
ypStage I	25 (21–37)	21 (16–31)	**0.020**
ypStage II	24 (20–30)	26 (19–33)	0.092
ypStage III	28 (19–34)	25 (19–36)	0.942
ypStage			**<0.001**
0	45 (36.9%)	0 (0%)	
I	39 (32.0%)	55 (26.3%)	
II	13 (10.7%)	67 (32.1%)	
III	25 (20.5%)	87 (41.6%)	
Circumferential resection margin			**0.013**
>1 mm	119 (97.5%)	189 (90.4%)	
≤1 mm	3 (2.5%)	20 (9.6%)	

Abbreviations: CRT = chemoradiotherapy. Data are presented as *n* (%), unless otherwise indicated.^a^ Median (interquartile range). Bold type indicates a significant value.

**Table 2:** Impact of different clinicopathologic factors on five-year outcome.

Variables	No. of Patients	5-Year DFS (%)	*p* Value	5-Year OS (%)	*p* Value
Age at diagnosis			0.084		**0.012**
≤61 years	161	79.7		91.5	
>61 years	170	72.0		82.6	
Sex			0.481		0.335
Male	229	75.5		86.3	
Female	102	75.8		88.0	
Clinical T classification			**0.040**		0.115
cT2	20	88.2		100	
cT3	278	75.5		85.6	
cT4	33	69.7		90.7	
Clinical N classification			**0.016**		0.054
cN0	50	90.4		95.1	
cN+	281	73.0		85.4	
Distance from anal verge			0.485		0.909
≤5 cm	197	73.8		87.1	
>5 cm	134	78.4		86.5	
Type of surgery			0.658		0.245
Sphincter-preserving surgery	293	75.8		87.2	
Abdominoperineal resection	38	75.0		84.1	
Interval completion of CRT to surgery			0.157		0.329
<42 days	35	68.3		82.5	
≥42 days	296	76.5		87.4	
Adjuvant chemotherapy			0.549		0.765
No	42	71.2		86.6	
Yes	289	76.3		86.9	
Lymphatic invasion			**0.001**		**0.001**
No	296	77.4		88.8	
Yes	33	57.6		68.6	
Venous invasion			**<0.001**		**0.002**
No	302	77.6		88.6	
Yes	27	51.6		63.9	
Perineural invasion			**<0.001**		**<0.001**
No	262	82.3		92.2	
Yes	67	48.3		63.3	
ypT classification			**<0.001**		**<0.001**
ypT0	50	96.0		100	
ypT1	22	94.1		100	
ypT2	91	81.2		93.6	
ypT3	164	64.4		77.4	
ypT4	4	50.0		75.0	
ypN classification			**<0.001**		**<0.001**
ypN0	219	85.3		94.7	
ypN1	87	59.2		73.7	
ypN2	25	47.7		58.5	
ypStage			**<0.001**		**<0.001**
0	45	97.8		100	
I	94	87.0		97.2	
II	80	75.9		88.7	
III	112	56.6		70.1	
Circumferential resection margin			**<0.001**		**<0.001**
>1 mm	308	77.8		88.4	
≤1 mm	23	47.1		65.7	
Tumor regression grade			**<0.001**		**<0.001**
4	50	96.0		100	
3	72	87.8		96.4	
2	154	69.2		83.4	
1	55	59.1		72.0	
0	0	–		–	
Grouped tumor regression grade			**<0.001**		**<0.001**
3/4	122	91.3		98.0	
1/2	209	66.6		80.6	

Abbreviations: CRT = chemoradiotherapy, DFS = disease-free survival, OS = overall survival. Bold type indicates a significant value.

**Table 3:** Multivariate analysis for prognostic factors.

Variables	Disease-Free Survival		Overall Survival	
	Hazard Ratio (95% CI)	*p* Value	Hazard Ratio (95% CI)	*p* Value
Grouped TRG				
1/2	Ref		Ref	
3/4	0.35 (0.19–0.66)	**0.001**	0.29 (0.10–0.84)	**0.023**
ypN classification				
ypN0	Ref		Ref	
ypN1	2.33 (1.42–3.83)	**0.001**	1.77 (0.88–3.56)	0.109
ypN2	3.71 (1.93–7.14)	**<0.001**	3.21 (1.40–7.35)	**0.006**
Perineural invasion				
No	Ref		Ref	
Yes	2.60 (1.63–4.14)	**<0.001**	2.26 (1.21–4.23)	**0.011**
Age	—	—	1.04 (1.01–1.07)	**0.008**
ypT classification				
ypT0–2			Ref	
ypT3–4	—	—	2.08 (0.89–4.85)	0.091
CRM				
>1 mm			Ref	
≤1 mm	—	—	2.08 (0.95–4.56)	0.067

Abbreviations: TRG = tumor regression grade, CRM = circumferential resection margin. Bold type indicates a significant value.

**Table 4:** New modified staging system.

**modified Stage 0**	ypStage 0–I & TRG 3/4, ypStage II & TRG 3/4
**modified Stage I**	ypStage 0–I & TRG 1/2
**modified Stage II**	ypStage II & TRG 1/2, ypStage III & TRG 3/4
**modified Stage III**	ypStage III & TRG 1/2
